# Trends and determinants for early initiation of and exclusive breastfeeding under six months in Vietnam: results from the Multiple Indicator Cluster Surveys, 2000–2011

**DOI:** 10.3402/gha.v9.29433

**Published:** 2016-02-29

**Authors:** Quyen Thi-Tu Bui, Hwa-Young Lee, Anh Thi-Kim Le, Do Van Dung, Lan Thi-Hoang Vu

**Affiliations:** 1 Department of Epidemiology and Biostatistics, The Hanoi School of Public Health, Hanoi, Vietnam; 2 JW Lee Center for Global Medicine, Seoul National University College of Medicine, Seoul, South Korea; 3 Ho Chi Minh City University of Medicine and Pharmacy, Ho Chi Minh City, Vietnam

**Keywords:** breastfeeding, multilevel analysis, MICS, Vietnam

## Abstract

**Background:**

There is strong evidence that breastfeeding (BF) significantly benefits mothers and infants in various ways. Yet the proportion of breastfed babies in Vietnam is low and continues to decline. This study fills an important evidence gap in BF practices in Vietnam.

**Objective:**

This paper examines the trend of early initiation of BF and exclusive BF from 2000 to 2011 in Vietnam and explores the determinants at individual and contextual levels.

**Design:**

Data from three waves of the Multiple Indicator Cluster Survey were combined to estimate crude and adjusted trends over time for two outcomes – early initiation of BF and exclusive BF. Three-level logistic regressions were fitted to examine the impacts of both individual and contextual characteristics on early initiation of BF and exclusive BF in the 2011 data.

**Results:**

Both types of BF showed a decreasing trend over time after controlling for individual-level characteristics but this trend was more evident for early initiation of BF. Apart from child's age, individual-level characteristics were not significant predictors of the BF outcomes, but provincial characteristics had a strong association. When controlling for individual-level characteristics, mothers living in provinces with a higher percentage of mothers with more than three children were more likely to have initiated early BF (odds ratio [OR]: 1.06; confidence interval [CI]: 1.02–1.11) but less likely to exclusively breastfeed their babies (OR: 0.94; CI: 0.88–1.01). Mothers living in areas with a higher poverty rate were more likely to breastfeed exclusively (OR: 1.07; CI: 1.02–1.13), and those who delivered by Caesarean section were less likely to initiate early BF.

**Conclusions:**

Our results suggest that environmental factors are becoming more important for determining BF practices in Vietnam. Intervention programs should therefore not only consider individual factors, but should also consider the potential impact of contextual factors on BF practices.

## Introduction

Breastfeeding (BF) benefits newborns and mothers in various ways, such as by providing nutrients for facilitating growth or boosting the immunity of babies. Research shows that BF helped reduce about 804,000 deaths among children under five in 2011 – equivalent to nearly 11.6% of deaths among under-five children worldwide (1). It has also been estimated that 44 million disability-adjusted life years (DALYs), which is 10% of DALYs in children under five, were attributed to not receiving BF (1). For these reasons the World Health Organization and the United Nations International Children's Emergency Fund (2) strongly recommend feeding breast milk to infants, especially in the first 6 months after birth.

However, rates of BF, including early initiation of BF, defined as being within the first hour after birth, and feeding only breast milk during the first 6 months of life (exclusive BF), have not increased in recent years. Developing countries such as Vietnam have made slow progress in this area (3, 4). According to data presented by Thu et al. in 2012 (5), the proportion of both early initiation of BF and exclusive BF in Vietnam was below 50% in babies born between 2008 and 2010, although the overall proportion of BF in the population reached 98% (6–8). In addition, it is of concern that rates of BF in Vietnam have been trending downwards over the recent years.

A few studies have been published on the determinants of early initiation of BF and exclusive BF in Vietnam. Almroth et al. (9) interviewed 118 mothers, fathers, and health workers in Vietnam to assess views on the feasibility of exclusive BF. Poor understanding and appreciation by mothers about the benefits of BF were cited as the main reasons for low BF rates. Duong et al. (10) explored the determinants of early initiation of BF in rural Vietnam using data collected in 2002–2003 and found that mothers’ education level, mothers’ feelings or beliefs about BF, and fathers’ occupation were significant factors that influenced early initiation of BF.

On the other hand, a systematic review of BF practices conducted in 2011 suggested that there have been many barriers to BF practices in Vietnam (4). They included individual-level factors associated with mothers and families and (or) ecological factors that referred to community and society. Another study on BF suggested that contextual factors can have important effects on health behaviors and health outcomes (11, 12). However, most of the studies on BF in Vietnam have focused on individual-level factors. Those few studies that have considered contextual factors have used qualitative methods or are outdated (9, 13, 14). There is a need to explore the impact of contextual factors on BF in order to have better understanding of the determinants of BF.

This paper has two main aims. The first is to examine the trends in early initiation of BF and exclusive BF in Vietnamese mothers in 2000–2011, while adjusting for individual-level factors. The second goal is to examine the association between both individual- and contextual-level factors and 1) early initiation of BF and 2) exclusive BF in Vietnam.

## Methods

### Data sources

All individual-level variables were derived from Multiple Indicator Cluster Survey (MICS) in Vietnam. The 2009 Vietnam Population and Housing Census (VPHC) (15) and the 2008 Vietnam Living Standard Survey (VLSS) were used as the source of provincial-level variables (16).

The MICS in Vietnam was conducted by the General Statistics Office in collaboration with the Ministry of Health and the Ministry of Labor, Invalids and Social Affairs with the purpose of collecting information on issues affecting the health, development, and living conditions of Vietnamese women and children. Currently, the data from 3 years have been released: 2000 (Wave 2), 2006 (Wave 3), and 2011 (Wave 4). All the results and datasets of the MICS are in the public domain and can be accessed via the related website (17).

The 2008 VLSS (16) was conducted nationwide for the purpose of systematic monitoring and supervision of living standards in different population groups in Vietnam. The sample consists of 45,945 households (36,756 households in the income survey and 9,189 households surveyed on both income and expenditure) within 3,063 communes/wards that were representative at the national, regional, urban, rural, and provincial levels. The survey collected information during two periods in 2008 through face-to-face interviews with household heads and key commune officials in the sample enumeration areas (16).

The 2009 VPHC (15) was the fourth population census and the third housing census implemented in Vietnam since the nation was reunified in 1975. The census aimed to collect basic data on population and housing for the entire territory of the Socialist Republic of Vietnam and to provide data for research about population trends and housing developments, at both the national and local levels. It responded to the country's need for information: to assess the implementation of the socioeconomic development plans covering 2001 to 2010; for developing the socioeconomic development plans 2011 to 2020, and to monitor Vietnam's performance on the United Nations Millennium Development Goals, to which the Vietnamese Government is committed (18).

### Study design

The MICS was conducted at the national level, for urban and rural areas, and for six Vietnamese regions (Red River Delta, Mekong River Delta, Northern Midland and Mountain area, North Central and Central Coastal area, South East, and Central Highlands). The urban and rural areas within each region were identified as the main sampling strata. The sample was selected in two stages. Within each stratum, a certain number of census enumeration areas were selected using the probability proportional to size. A systematic sample of 20 households was drawn from each enumeration area (6–8). Each survey contained three well-structured separate datasets with information on households, women aged 15–49 years, and children under five living in Vietnam.

### Measures

#### Outcome variables

The two binary outcomes of interest are ‘early initiation of BF’ and ‘exclusive BF’ (yes/no). ‘Early initiation of BF’ refers to whether or not the child was breastfed within the first hour after birth. This information was only available in the 2006 and 2011 MICS women's datasets. Mothers with children under 2 years old at the time of the survey were asked when they started BF their child after delivering. Information for the exclusive BF outcome was collected from the 2000, 2006, and 2011 MICS children's datasets. This indicates whether infants received only breast milk (and possibly vitamins, mineral supplements, or medicine) during the first 6 months of their lives. Information about specific kinds of foods, fruits, and fluids the child was given during the 24 h prior to the interview was reported either by mothers with an infant under 6 months of age, or else by caregivers responsible for the infant's consumption of food and fluids within the day prior to interview.

#### Explanatory variables

The selection of explanatory variables was informed by the literature. Factors theoretically and empirically associated with HIV/AIDS outcomes were identified by reviewing the literature. Among the possible candidates for the explanatory variables, we selected those for which data were available in both 2006 and 2011 for the analysis of early initiation of BF and in all 3 years (2000, 2006, and 2011) for the analysis of exclusive BF.

##### Individual-level factors

Information about early initiation of BF and exclusive BF was extracted from two distinct datasets, with different variables describing women's and children's characteristics. For the analysis of early initiation of BF, we included demographic variables (mother's age, living area, and ethnicity), socioeconomic variables (mother's education and household wealth), and delivery-related variables such as size of child at birth and place of delivery. For the analysis of exclusive BF, we included demographic variables such as child's age, sex, ethnicity, living area, and socioeconomic variables such as mother's education and household wealth.

Vietnam is a multiethnic country with over 50 distinct ethnic groups. The Kinh is the ethnic majority, accounting for about 86% of the population, and the standard of living between the Kinh and the other minorities is quite different. Therefore, mothers’ ethnicity was classified into two groups, Kinh (or Hoa) and non-Kinh. The Chinese ethnic minority Hoa was grouped with the Kinh ethnic majority because both groups have similar living standards.

For the socioeconomic status variables, mother's educational level was grouped into five categories: no education (had never been to school), primary (Grades 1 to 5), lower secondary (Grades 6–9), upper secondary (Grades 10–12), and tertiary (professional school, college, or above university). A household wealth status variable captured underlying long-term wealth based on ownership of consumer goods, dwelling characteristics, and water and sanitation. Principal component analysis was used to derive wealth scores, which were used to rank households into wealth quintiles from the poorest to the wealthiest (17).

The size of the child at birth was based on the mother's assessment. This was regrouped from five categories (very small, smaller than average, average, larger than average, very large) into three categories: smaller than average, average, and larger than average.

##### Provincial-level factors

The provincial-level variables included the percentage of poor households in each province according to Vietnamese standards.^1^ This information was extracted from the 2008 VLSS. Information on the percentage of women aged 15–49 who had more than three children was derived from the VPHC 2009 (15).

### Data analysis

Trends in exclusive BF were analyzed across the three waves of the MICS dataset on children. Trends in early initiation of BF were analyzed across the two waves of the MICS dataset on women. Survey year was identified by a ‘wave’ variable (Wave 2, 2000; Wave 3, 2006; and Wave 4, 2011). Logistic regressions described crude and adjusted association between survey year (wave) and each BF outcome.

In order to account for the hierarchical structure of the data, where individuals (Level 1) were nested in provinces (Level 2), which were again nested within regions (Level 3), and in order to identify determinants at multiple levels, three-level random intercept logistic modeling was undertaken using the fourth (most recent) wave of the MICS, which took place in 2011 (19). A series of three models were fitted. Model 1, a three-level empty model, was fitted without including any explanatory variables. Variation in the probability of early initiation of BF and exclusive BF was partitioned across the three levels. In Model 2, the sociodemographic model, only individual-level factors were included. The full model, Model 3, expanded upon Model 2 by adding provincial-level factors.

In order to further investigate the determinants for the early initiation of BF, multilevel logistic analysis was undertaken in the 2011 women's dataset with the inclusion of a delivery mode variable (Table 7). This variable was not included as a predictor variable in the analysis for examining trends in BF because the variable for delivery mode was only available in the 2011 MICS. Delivery mode was assessed by asking if respondents delivered the baby by Caesarian section or not. The variable for place of birth was dropped because of colinearity.

By simultaneously considering both individual and geographical-level variables, Model 3 enabled us to estimate whether the determinants for each BF outcome were compositional, contextual, or both.

We restricted our study sample to those who had non-missing responses on all variables. For each model, the results of the fixed effects (measures of association) were shown as odds ratios (OR) with 95% confidence intervals (CIs). The results of random effects (measures of variation) were presented as the variance and intraclass correlation coefficient (ICC) at the regional and provincial levels.

The ICC for individuals within the same province, but in different regions, was calculated as follows:

$$\frac{\text{Varianc}{\text{e}}_{\text{province}}}{\text{Varianc}{\text{e}}_{\text{province}}+\text{Varianc}{\text{e}}_{\text{region}}+\pi^{\text{2}}/3}$$

The ICC for individuals within the same region was calculated as follows:

$$\frac{\text{Varianc}{\text{e}}_{\text{province}}+\text{Varianc}{\text{e}}_{\text{region}}}{\text{Varianc}{\text{e}}_{\text{province}}+\text{Varianc}{\text{e}}_{\text{region}}+\pi^{\text{2}}/\text{3}}$$

A *p*-value<0.05 was regarded as statistically significant. Statistical analyses were performed using STATA 13.0.

### Ethical considerations

This paper was based on secondary data from the MICS, VPHC, and VLSS with all identifying information removed. The survey obtained informed consent from the mothers before administering survey questionnaires. All information in the original dataset was collected confidentially (6, 7, 15).

## Results

### Derivation of the study samples

**Fig. 1:**
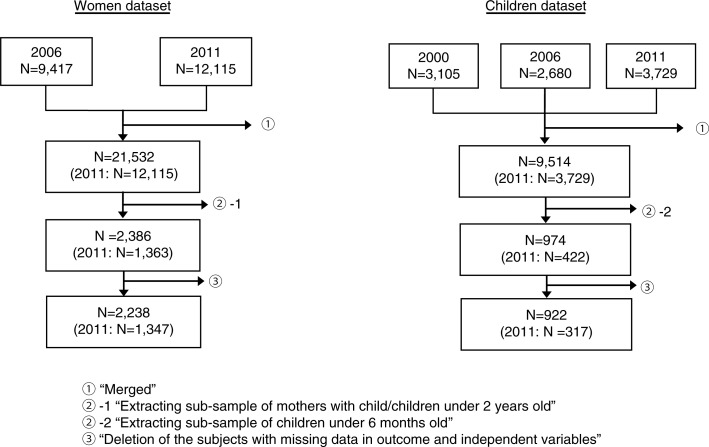
Derivation of study samples in each dataset.

Figure 1 shows the derivation of study samples from each dataset (i.e. women and children). After applying the exclusion criteria, the study samples comprised 1,347 and 317 observations for the analyses of women and children, respectively.

### Sample characteristics and trends in each type of BF

**Fig. 2:**
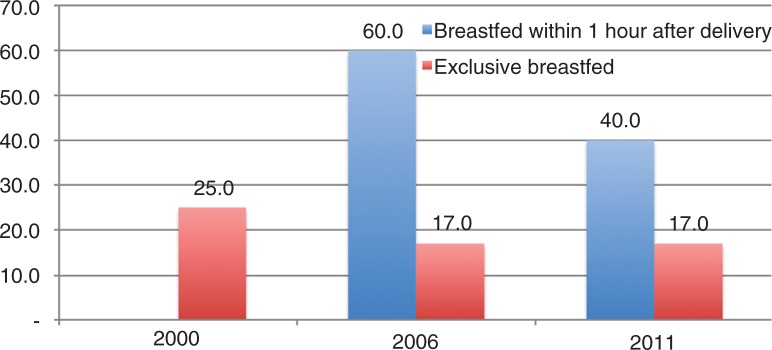
Trends in early initiation of breastfeeding and exclusive breastfeeding in Vietnam.

Figure 2 shows decreasing trends in percentages for early initiation of BF between 2006 and 2011 and exclusive BF between 2000 and 2011 in Vietnam. (As previously mentioned, information for early initiation of BF was not available in the 2000 data.) Early initiation of BF showed a big drop during 2006–2011, while exclusive BF was constant during the same period after declining by about 8% during 2000–2006.

Tables 1 and 2 present the prevalence of early initiation of BF and exclusive BF by individual characteristics. Prevalence of early initiation of BF fell in all groups between 2006 and 2011, while in the same years the prevalence of exclusive BF increased for female children, children living in urban areas, non-Kinh mothers, mothers with no education, and mothers in the richest and second poorest quintiles. When we look at the 2011 data, the prevalence of early initiation of BF and the prevalence of exclusive BF were both noticeably higher in mothers whose ethnicity were non-Kinh, belonged to the poorest wealth quintile, and had no education.

**Table 1:** Descriptive statistics for mothers with children under 2 years old (dataset for early initiation of BF)

	2006 (*N*=891)			2011 (*N*=1,347)		
		
	Total *N*	Early initiation of BF (*N*)	%	Total *N*	Early initiation of BF (*N*)	%
Mother's age (mean±SD)	27.71±5.91			27.45±5.54		
Ethnicity of mother						
Non-Kinh	191	132	69.1	281	154	54.8
Kinh	700	405	57.9	1,066	381	35.7
Area						
Urban	218	121	55.5	534	173	32.4
Rural	673	416	61.8	813	362	44.5
Wealth index						
Richest	166	88	53.0	301	107	35.5
Fourth	192	115	59.9	266	80	30.1
Middle	191	120	62.8	237	83	35.0
Second	167	94	56.3	221	94	42.5
Poorest	175	120	68.6	322	171	53.1
Education of mother						
Tertiary	122	77	63.1	290	107	36.9
Upper secondary	114	67	58.8	282	105	37.2
Lower secondary	436	271	62.2	487	196	40.2
Primary	218	121	55.5	199	77	38.7
No education	1	1	100.0	89	50	56.2
Marital status						
Living with spouse	865	521	60.2	1,326	527	39.7
Windowed/divorced/separated	13	10	76.9	14	7	50.0
Never married	13	6	46.2	7	1	14.3
Size of child at birth						
Smaller than average	150	95	63.3	149	55	36.9
Average	617	380	61.6	1,071	434	40.5
Larger than average	124	62	50.0	127	46	36.2
Place of delivery						
At home	147	92	62.6	135	83	61.5
Government hospital	486	273	56.2	933	326	34.9
Other public health facility	221	153	69.2	213	100	46.9
Private health sector	37	19	51.4	66	26	39.4

BF, breastfeeding

**Table 2:** Descriptive statistics for mothers with children under 6 months old (dataset for exclusive BF)

	2001 (*N*=291)			2006 (*N*=260)			2011 (*N*=371)		
			
	Total *N*	Exclusive BF (*N*)	%	Total *N*	Exclusive BF (*N*)	%	Total *N*	Exclusive BF (*N*)	%
Child's age (months)	3.51±1.91			3.12±1.79			3.06±1.92		
Ethnicity of mother									
Non-Kinh	94	23	24.5	89	24	27.0	86	24	27.9
Kinh	197	34	17.3	171	20	11.7	285	30	10.5
Area									
Urban	47	8	17.0	54	4	7.4	136	14	10.3
Rural	244	49	20.1	206	40	19.4	235	40	17.0
Sex									
Female	155	33	21.3	128	19	14.8	183	32	17.5
Male	136	24	17.6	132	25	18.9	188	22	11.7
Wealth index									
Richest	35	5	14.3	39	2	5.1	78	7	9.0
Fourth	47	11	23.4	48	7	14.6	65	4	6.2
Middle	46	6	13.0	47	9	19.1	75	11	14.7
Second	61	12	19.7	42	3	7.1	57	7	12.3
Poorest	102	23	22.5	84	23	27.4	96	25	26.0
Education of mother									
Tertiary	15	1	6.7	37	5	9.1	84	11	13.1
Upper secondary	34	8	23.5	55	26	18.4	86	15	17.4
Lower secondary	97	15	15.5	0	0	0	111	13	11.7
Primary	81	20	24.7	141	5	18.5	64	7	10.9
No education	64	13	20.3	27	8	21.6	26	8	30.8

Table 3 shows the results of the crude and adjusted logistic regressions of associations between survey year and early initiation of BF. In the crude model, the odds of early initiation of BF in 2011 were significant (*p<*0.001) and almost 60% lower than the reference year, 2006 (OR: 0.44; CI: 0.37–0.52). When adjusting for individual characteristics, the odds were similar (AOR: 0.42; CI: 0.31–0.52).

**Table 3:** Crude and multivariable logistic regression for the early initiation of BF in Vietnam, 2006 and 2011 (*N*=2,238)

		Crude		Adjusted	
			
Factor	Proportion	OR	95% CI	OR	95% CI
Year					
2006	59.9	Ref		Ref	
2011	39.7	***0.44	(0.37–0.52)	***0.42	(0.35–0.51)
Mother's age	–			1.00	(0.98–1.01)
Ethnicity of mother					
Non-Kinh	60.6			Ref	
Kinh	44.4			**0.68	(0.51–0.91)
Area					
Urban	52.8			Ref	
Rural	39.1			0.81	(0.65–1.02)
Wealth index					
Richest	42.0			Ref	
Fourth	42.2			0.92	(0.69–1.23)
Middle	47.0			1.05	(0.77–1.44)
Second	48.4			**1.08	(0.77–1.51)
Poorest	59.2			1.39	(0.95–2.03)
Education of mother					
Tertiary	44.6			Ref	
Upper secondary	43.3			0.85	(0.63–1.14)
Lower secondary	50.7			0.85	(0.65–1.12)
Primary	47.4			0.60	(0.43–0.84)
No education	56.7			0.86	(0.50–1.47)
Marital status					
Living with spouse	48.4			Ref	
Widowed/divorced/separated	58.6			1.62	(0.71–3.70)
Never married	35.0			0.43	(0.16–1.15)
Size of child at birth					
Smaller than average	50.2			Ref	
Average	49.0			0.95	(0.74–1.24)
Larger than average	42.4			*0.68	(0.47–0.96)
Place of delivery					
At home	61.9			Ref	
Government hospital	42.1			0.74	(0.53–1.04)
Other public health facility	58.6			1.09	(0.77–1.54)
Private health sector	42.9			0.83	(0.50–1.37)

* *p*<0.05

** *p<*0.01

*** *p*<0.001. OR, odds ratio; CI, confidence interval.

Table 4 shows the results of the crude and adjusted logistic regressions of associations between survey year and exclusive BF. In the crude model, the odds of exclusive BF in 2006 (OR: 0.84; CI: 0.54–1.29) or 2011 (OR: 0.70; CI: 0.46–1.50) were not significantly different from those in 2000. However, when adjusting for individual characteristics, the odds of exclusive BF were significantly lower in 2006 (AOR: 0.56; CI: 0.32–0.96) and 2011 (AOR: 0.58; CI: 0.36–0.94) compared with those in 2000.

**Table 4:** Crude and multivariable logistic regression for exclusive BF in Vietnam, 2000, 2006 and 2011 (*N*=922)

		Crude		Adjusted	
			
Factor	Proportion	OR	95% CI	OR	95% CI
Year					
2000	24.7				
2006	17.0	0.84	(0.54–1.29)	*0.56	*(0.32–0.96)
2011	16.9	0.70	(0.46–1.50)	*0.58	*(0.36–0.94)
Child's age (months)				0.62	***(0.56–0.70)
Ethnicity					
Non-Kinh	26.4			Ref	
Kinh	12.9			0.44	**(0.26–0,74)
Area					
Urban	18.8			Ref	
Rural	11.0			0.67	(0.37–1.23)
Sex					
Female	20.6			Ref	
Male	17.7			0.80	(0.55–1.17)
Wealth index					
Richest	10.4			Ref	
Fourth	15.3			1.35	(0.61–3.01)
Middle	17.9			1.42	(0.61–3.29)
Second	15.7			1.04	(0.42–2.56)
Poorest	29.0			2.01	(0.81–5.00)
Education of mother					
Tertiary	11.0			Ref	
Upper secondary	31.6			1.27	(0.65–2.49)
Lower secondary	18.1			0.80	(0.37–1.72)
Primary	20.7			0.88	(0.40–1.97)
No education	18.7			0.63	(0.26–1.56)

* *p*<0.05

** *p*<0.01

*** *p*<0.001.

Tables 5 and 6 present the adjusted odds ratios for women and children who had breastfed (or been breastfed) versus not breastfed (or not been breastfed) in 2011 from the three models. In the null model, the proportion explained by between-region variance of the total variance (the ICC) in the early initiation of BF was quite small (ICC_region_=2.2%). By contrast, a larger proportion of the total variance explained by between-province variance was identified (ICC_province_=18.8%).

**Table 5:** Three-level logistic regression for determinants of the early initiation of BF, 2011 (*n*=1,347)

Factor		Empty model	Model 1 OR (95% CI)	Model 2 OR (95% CI)
Fixed effects				
Individual level				
Demographic factors	Mother's age (years)		0.99 (0.97–1.01)	0.99 (0.97–1.01)
	Ethnicity			
	Non-Kinh	Ref		
	Kinh		0.88 (0.56–1.40)	0.92 (0.58–1.47)
	Area			
	Urban	Ref		
	Rural		0.76 (0.57–1.03)	0.75 (0.56–1.02)
Socioeconomic factors	Wealth index			
	Richest	Ref		
	Fourth		0.63 (0.42–0.93)	0.62 (0.41–0.92)
	Middle		0.74 (0.48–1.15)	0.73 (0.47–1.15)
	Second		0.96 (0.59–1.55)	0.96 (0.59–1.57)
	Poorest		0.88 (0.51–1.53)	0.85 (0.49–1.48)
	Education of mother			
	Tertiary	Ref		
	Upper secondary		0.95 (0.65–1.40)	0.96 (0.65–1.41)
	Lower secondary		1.09 (0.75–1.59)	1.06 (0.72–1.55)
	Primary		0.89 (0.55–1.44)	0.86 (0.52–1.41)
	No education		0.92 (0.45–1.86)	0.84 (0.41–1.73)
	Marital status			
	Living with spouse	Ref		
	Widowed/divorced/separated		1.73 (0.55–5.46)	1.74 (0.55–5.56)
	Never married		0.33 (0.04–2.85)	0.35 (0.04–3.04)
Delivery-related factors	Size of child at birth			
	Smaller than average	Ref		
	Average		1.02 (0.69–1.50)	1.05 (0.72–1.55)
	Larger than average		0.82 (0.48–1.40)	0.83 (0.48–1.42)
	Place of delivery			
	At home	Ref		
	Government hospital		0.71 (0.40–1.27)	0.78 (0.43–1.39)
	Other public health facility		0.91 (0.51–1.63)	1.00 (0.55–1.80)
	Private health sector		0.82 (0.38–1.73)	0.86 (0.40–1.87)
	Prenatal care visits			
	Yes			
	No		0.81 (0.43–1.52)	0.85 (0.40–1.87)
Provincial level				
% women with three children or more				**1.06 (1.02–1.11)
% poverty				0.99 (0.96–1.02)
Random effects				
Regional (variance)		0.089	0.038	0.051
Provincial (variance)		0.673	0.617	0.671
ICC_region_ (%)		2.2	1.0	1.3
ICC_province_ (%)		18.8	16.6	18.0

*p<0.05

** *p*<0.01

***p<0.001. ICC, intraclass correlation coefficient.

**Table 6:** Three-level logistic regression for the determinants of exclusive BF, 2011 (*N*=371)

Factor		Empty model	Model 1 OR (95% CI)	Model 2 OR (95% CI)
Fixed effects				
Individual-level factors				
Demographic factors	Child's age (months)		***0.58 (0.47–0.71)	***0.57 (0.46–0.70)
	Ethnicity			
	Non-Kinh	Ref		
	Kinh		0.49 (0.17–1.36)	0.59 (0.21–1.72)
	Area			
	Urban	Ref		
	Rural		0.86 (0.35–2.13)	0.90 (0.36–2.27)
	Sex			
	Female	Ref		
	Male		0.50 (0.24–1.02)	0.51 (0.25–1.05)
Socioeconomic factors	Wealth index			
	Richest	Ref		
	Fourth		0.70 (0.17–2.93)	0.74 (0.18–3.06)
	Middle		1.56 (0.43–5.62)	1.52 (0.42–5.51)
	Second		1.42 (0.31–6.22)	1.11 (0.23–5.28)
	Poorest		2.69 (0.53–13.58)	2.24 (0.43–11.77)
	Education of mother			
	Tertiary	Ref		
	Upper secondary		1.21 (0.41–3.58)	1.38 (0.46–4.16)
	Lower secondary		0.72 (0.22–2.33)	0.83 (0.25–2.74)
	Primary		0.49 (0.12–2.03)	0.58 (0.13–2.50)
	No education		0.91 (0.18–4.74)	1.11 (0.20–6.17)
Provincial-level factors				
% women with three children or more				*0.94 (0.88–1.01)
% poverty				*1.07 (1.02–1.13)
Random effects				
Regional variance		0.68	0.41	0.10
Provincial variance		0.01	0.00	0.14
ICC – region		17.1	11.1	2.8
ICC – province		17.3	11.1	6.6

* *p*<0.05

**p<0.01

*** *p*<0.001.

In Model 1 as well as in Model 2, the individual-level variables were not significant in association with early initiation of BF (*p*>0.05). In the full model (Model 2), the proportion of women with more than three children was significantly associated with early initiation of BF (*p<*0.01). When the proportion of women with more than three children in provinces increased by 1%, the odds of the mothers initiating BF within 1 h after delivery increased by 6% (CI: 2–11%) (see Table 5).

Most of the individual-level variance in the early initiation of BF was attributed to provincial-level variance (ICC_province_=18.0%). Even after all the individual- and provincial-level variables were included, there was still considerable between-region and between-province variation in the full model.

The results of the multilevel logistic regression with exclusive BF under 6 months as the outcome differed from the results of early initiation of BF analysis. In the null model, the proportion of between-region variance of the total variance was larger than for the early initiation of BF (ICC_region_=2.2%, 17.01%, respectively). In Models 1 and 2, of the sociodemographic variables, only child's age was significant in association with exclusive BF. For a 1-month increase in age, infants had a 0.57 times lower odds of receiving exclusive BF (*p<*0.001). In the full model (Model 2), significant associations were found between two provincial-level variables and exclusive BF. Specifically, when the proportion of poverty increased by 1% in a province, infants under 6 months of age had a 1.07 times higher odds of being exclusively breastfed during the first 6 months of their lives (*p<*0.05; CI: 1.02–1.13). When the proportion of women with more than three children increased by 1% in a province, infants under 6 months of age had 0.94 times odds of being exclusively breastfed for 6 months after their birth (*p<*0.05; CI: 0.88–1.01).

Caesarean section was a major explanatory variable in the multilevel regression model for the early initiation of BF. The odds of mothers who underwent Caesarean section in their delivery breastfeeding their babies within the first hour after delivery were 90% lower than for other mothers (OR: 0.10; *p<*0.001; CI: 0.06–0.17) (see Table 7).

**Table 7:** Analysis for determinants of the early initiation of BF, 2011 (including delivery mode variable) (*N*=1,029)

Factors		Empty model	Model 1 OR (95% CI)	Model 2 OR (95% CI)
Fixed effects				
Individual level				
Demographic factors	Mother's age (years)		1.02 (0.99–1.05)	1.02 (0.99–1.05)
	Ethnicity			
	Non-Kinh	Ref		
	Kinh		0.88 (0.52–1.49)	0.94 (0.55–1.60)
	Area			
	Urban	Ref		
	Rural		0.75 (0.53–1.06)	0.73 (0.52–1.04)
Socioeconomic factors	Wealth index			
	Richest	Ref		
	Fourth		†0.63 (0.39–1.00)	*0.61 (0.38–0.98)
	Middle		0.71 (0.43–1.18)	0.69 (0.41–1.15)
	Second		0.82 (0.47–1.43)	0.79 (0.45–1.39)
	Poorest		0.87 (0.46–1.53)	0.80 (0.42–1.51)
	Education of mother			
	Tertiary	Ref		
	Upper secondary		0.82 (0.53–1.28)	0.83 (0.53–1.29)
	Lower secondary		0.87 (0.56–1.35)	0.87 (0.56–1.36)
	Primary		0.61 (0.34–1.09)	0.61 (0.34–1.10)
	No education		0.79 (0.30–2.08)	0.76 (0.28–2.02)
	Marital status			
	Living with spouse	Ref		
	Widowed/divorced/separated		1.53 (0.35–6.56)	1.60 (0.37–6.87)
	Never married		0.53 (0.05–5.56)	0.51 (0.05–5.31)
Delivery-related factors	Size of child at birth			
	Smaller than average	Ref		
	Average		0.84 (0.53–1.35)	0.86 (0.54–1.37)
	Larger than average		0.59 (0.30–1.14)	0.58 (0.30–1.13)
	Delivery mode			
	No Caesarian section	Ref		
	Caesarian section		***0.10 (0.06–0.17)	***0.10 (0.06–0.17)
	Visit to prenatal care			
	Yes			
	No		1.15 (0.43–3.08)	1.14 (0.42–3.04)
Provincial level				
% women with three children or more				1.03 (0.98–1.04)
% poverty				1.01 (0.98–1.05)
Random effects				
Regional (variance)		0.051	0.046	0.028
Provincial (variance)		0.440	0.537	0.527
ICC_region_ (%)		1.4	1.2	0.7
ICC_province_ (%)		13.0	15.1	14.4

* *p*<0.05

**p<0.01

*** *p*<0.001

† marginally significant at *p*=0.05.

## Discussion

The aims of this study were twofold. First, we investigated changes in the early initiation of BF across two time points (2006 and 2011) and exclusive BF across three time points from 2000 to 2011. Second, we examined factors associated with early initiation of BF and exclusive BF using data from the 2011 MICS.

Our findings from the first analysis suggest that both rates for the early initiation of BF and exclusive BF decreased over time. This is an unlikely result in developed countries, but consistent with previous studies in Vietnam and other developing countries (5, 20–23). Specifically, the early initiation of BF in our study sample fell sharply between 2006 and 2011 from 59.9% in 2006 to 39.7% in 2011, and exclusive BF fell from 24.7 to 16.9% during the same period. Not only did exclusive BF rates fall, but absolute rates were also low – about 24.7% in 2000 and less than 16.9% in 2011. This is not a new finding; previous studies have already reported very low rates of exclusive BF in Vietnam (10, 24, 25). Among 14 countries in the Asia Pacific region, Vietnam was ranked as the lowest in terms of rate of exclusive BF (26). Our results are consistent with this finding.

The declining BF trend for the Vietnamese women in the surveys could be attributed to either the individual characteristics of the respondents or to extrinsic factors. However, an increasing BF trend was evident, even after controlling for important individual factors in the multivariable logistic regression. Therefore, parenting-related extrinsic factors may be contributing to this decline.

Caesarean section is one example. The fact that delivering babies through Caesarean section in Vietnam has increased in recent years (27) may be one of the environmental factors affecting BF practice. According to Lumbiganon et al., the proportion of Caesarean sections undertaken in Vietnam reached 36% in 2010, putting Vietnam among the countries with the highest Caesarean section rates in the world (28). Mothers who undergo a Caesarean section are separated from their babies for usually more than 1 h after birth, this being the best time for the early initiation of BF. Even if mothers can be with their babies after surgery, many refuse BF because they fear side effects from the medication that they received during their surgical procedure (29). This might therefore be a discouraging factor for the early initiation of BF (22, 29). Another environmental factor that may deter BF is the significant growth in the infant formula market during the last 15 years. Infant formula companies sometimes mislead consumers with exaggerated advertisements about the benefits of infant formula and even give false information that infant formula is better than breast milk. This type of aggressive marketing may affect mothers’ BF behavior (10, 24).

There were some notable findings from the analyses of factors associated with the early initiation of BF and exclusive BF. First, unlike many previous studies showing that individual socioeconomic and demographic factors such as mothers’ level of education, wealth, or residence area were associated with BF (5, 10, 24), we did not find a significant association between individual-level maternal factors and the early initiation of BF or exclusive BF. This suggests that mothers’ individual characteristics may no longer be as important in determining BF outcomes. In other words, education may not be as important for the uptake of knowledge and information about the benefits of BF as has been assumed.

Second, our study instead found an association between contextual factors (i.e. factors at the provincial level) and both BF outcomes. First, women living in provinces composed of people with lower levels of economic status (i.e. higher provincial poverty rates) seemed to have higher odds of exclusive BF, although this association was not significant for the early initiation of BF. There are a few possible explanations for this finding. One is the difference in Caesarean section rate among the provinces. Usually, Caesarean section deliveries tend to be more prevalent in affluent provinces than in the poorer provinces (27). As already explained here, this delivery method delays the possible time for the first BF. This was seen in previous studies in Vietnam and China (5, 29) as well as in our analysis (Table 7). Mothers who missed the chance for the early initiation of BF might have more difficulties in maintaining exclusive BF afterwards for both physiological and psychological reasons.

Another possible explanation for the association between higher levels of poverty and higher levels of exclusive BF is that mothers in better-off provinces often have more opportunities for exposure to aggressive commercials from infant formula companies. These areas have a larger market for infant formula and are targeted by infant formula companies (30). Understandably, mothers in more developed or wealthy provinces and urban areas are more likely to be able to afford formula milk, compared with mothers in poorer areas (10).

Lastly, we found that mothers living in provinces with a higher percentage of women with more than three children were more likely to give their babies the first breast milk within the first hour, but they were less likely to continue with exclusive BF during the first 6 months after birth. Provinces with a high proportion of young children might pay more attention to community programs for encouraging BF. The activities in many such programs tend to focus on encouraging the initiation of BF, for example, stationing health workers in community health centers where they can help mothers to start BF and give information about BF practices. However, they do not always follow up to find out whether mothers continue BF during the 6 months after delivery. Some mothers do not have a strong motivation for continuing BF because they have other young children to take care of. These results suggest that we need to consider not only the environmental factors associated with socioeconomic conditions, but also factors that can be directly related to the mothers’ real life patterns.

### Strengths and limitations

Although this study is the first to explore the relationship between contextual factors and the early initiation of BF and exclusive BF in Vietnam using the MICS data, we acknowledge that there are limitations. First, although this study used all the possible information from the MICS data, a large number of observations were dropped, resulting in a fairly small sample. Accordingly it can be argued that our analysis did not have sufficient power for identifying potential differences. This also impacts the generalizability of the results.

Second, because all the information about BF experience relied on maternal recall of the events, we acknowledge possible recall bias. However as questions about exclusive BF required respondents to recall events during the 24 h prior to the interview, it can be reasonably assumed that recall bias was not a major issue here. It is, however, possible that questions about the early initiation of BF were affected by recall bias. Recall bias can be reduced by ensuring that respondents have enough time to reflect before answering the questions or by having them think through a sequence of events related to the event at the investigation phase. However, since we used a secondary dataset, this tactic was not possible.

Last, the use of secondary data did not allow us to analyze the effects of the various factors that might have influenced BF behavior, such as mother's knowledge about BF and the experience of utilizing BF counseling during prenatal care. Given these limitations, our findings need to be interpreted with some caution.

## Conclusion

The results of this study indicate that BF practice in Vietnam is not as heavily affected by individual factors as previously thought. Environmental factors are becoming more important in supporting the adoption of BF practices. Furthermore, our findings suggest that Caesarean section may be a major impediment to BF. Considering this, as well as other well-known negative aspects of Caesarean sections, we suggest that the procedure should be performed only when necessary. Lastly, our empirical findings support the need for the greater regulation of information on infant formula, especially where this can mislead mothers about the benefits of BF versus formula feeding. It is intended that this study will be a cornerstone for further research in this important area. The next steps are to build a stronger evidence base by more specifically examining the links between contextual factors and BF. This will help to inform policy makers as they develop effective intervention programs to promote BF and enhance the nutritional and health status of babies and ultimately children (1).

## Footnotes

1 According to Vietnamese standards, the poor are defined as those with an average monthly income of less than VND 4.8 million (US$225) in rural areas and VND 6 million (US$282) in urban areas.
